# The tomato RLK superfamily: phylogeny and functional predictions about the role of the LRRII-RLK subfamily in antiviral defense

**DOI:** 10.1186/1471-2229-12-229

**Published:** 2012-12-02

**Authors:** Tetsu Sakamoto, Michihito Deguchi, Otávio JB Brustolini, Anésia A Santos, Fabyano F Silva, Elizabeth PB Fontes

**Affiliations:** 1 National Institute of Science and Technology in Plant-Pest Interactions, Universidade Federal de Viçosa, 36570-000, Viçosa, MG, Brazil; 2 Departamento de Bioquímica e Biologia Molecular/BIOAGRO, Universidade Federal de Viçosa, 36570-000, Viçosa, MG, Brazil; 3 Departamento de Estatística, Universidade Federal de Viçosa, 36570-000, Viçosa, MG, Brazil

**Keywords:** Receptor-like kinase, NIK, NSP, BAK1, Virus infection, Protein-protein interaction, Functional divergence, Plant signaling, *Solanum lycopersicum*

## Abstract

**Background:**

Receptor-like kinases (RLKs) play key roles during development and in responses to the environment. Despite the relevance of the RLK family and the completion of the tomato genome sequencing, the tomato RLK family has not yet been characterized, and a framework for functional predictions of the members of the family is lacking.

**Results:**

To generate a complete list of all the members of the tomato RLK family, we performed a phylogenetic analysis using the Arabidopsis family as a template. A total of 647 RLKs were identified in the tomato genome, which were organized into the same subfamily clades as Arabidopsis RLKs. Only eight of 58 RLK subfamilies exhibited specific expansion/reduction compared to their Arabidopsis counterparts. We also characterized the LRRII-RLK family by phylogeny, genomic analysis, expression profile and interaction with the virulence factor from begomoviruses, the nuclear shuttle protein (NSP). The LRRII subfamily members from tomato and Arabidopsis were highly conserved in both sequence and structure. Nevertheless, the majority of the orthologous pairs did not display similar conservation in the gene expression profile, indicating that these orthologs may have diverged in function after speciation. Based on the fact that members of the Arabidopsis LRRII subfamily (AtNIK1, AtNIK2 and AtNIK3) interact with the begomovirus nuclear shuttle protein (NSP), we examined whether the tomato orthologs of *NIK*, *BAK1* and *NsAK* genes interact with NSP of *Tomato Yellow Spot Virus* (ToYSV). The tomato orthologs of NSP interactors, SlNIKs and SlNsAK, interacted specifically with NSP in yeast and displayed an expression pattern consistent with the pattern of geminivirus infection. In addition to suggesting a functional analogy between these phylogenetically classified orthologs, these results expand our previous observation that NSP-NIK interactions are neither virus-specific nor host-specific.

**Conclusions:**

The tomato RLK superfamily is made-up of 647 proteins that form a monophyletic tree with the Arabidopsis RLKs and is divided into 58 subfamilies. Few subfamilies have undergone expansion/reduction, and only six proteins were lineage-specific. Therefore, the tomato RLK family shares functional and structural conservation with Arabidopsis. For the LRRII-RLK members *SlNIK1* and *SlNIK3*, we observed functions analogous to those of their Arabidopsis counterparts with respect to protein-protein interactions and similar expression profiles, which predominated in tissues that support high efficiency of begomovirus infection. Therefore, NIK-mediated antiviral signaling is also likely to operate in tomato, suggesting that tomato NIKs may be good targets for engineering resistance against tomato-infecting begomoviruses.

## Background

Plant cells constantly react to multiple signals that come from the local environment, neighboring cells, or even from other organisms. Depending on the stimuli, plant cells may expand, divide, differentiate, synthesize compounds, prepare against pathogen infection, or induce necrosis [1]. To perceive and receive these signals, plant cells possess complex systems of transmembrane receptor proteins that facilitate communication between the intracellular environment and the outside world.

One of the largest groups of these receptors is the receptor-like kinase (RLK) superfamily, which contains over 600 members in Arabidopsis [2-4]. RLKs are structurally organized into an extracellular domain that can be highly divergent, followed by a transmembrane segment and a conserved intracellular serine/threonine kinase domain. Most RLKs are localized in the plasma membrane, although there are also RLK members that are found in the cytoplasm. In this case, RLKs do not possess either an extracellular region or a transmembrane domain and are called receptor-like cytoplasmic kinases (RLCKs). Analyses of Arabidopsis RLKs by structural comparison of their extracellular region and phylogenetic analysis of their kinase domain revealed that they can be divided into over 50 subfamilies [5].

Several distinct RLKs have been studied in the past decade, and a common theme that has emerged is that binding of a specific signal molecule to their extracellular domain is required to initiate a signal transduction cascade [6]. Generally, ligand-receptor interactions at the extracellular domain of RLKs initiate the propagation of the signal through the membrane by inducing a conformational change in the receptor kinase domain, which allows interactions with other RLKs resulting in homo- or heterodimers. Dimerized RLKs are then transphosphorylated by their cytoplasmic kinase domain, leading to both activation of the kinase and establishment of docking sites for phosphorylation of downstream phosphorylation targets [7,8]. This activation mechanism of plant RLKs is similar to that of signal transduction mediated by receptor tyrosine kinases in animal cells, which share a common origin with plant serine/threonine kinases [3].

Functional analysis of RLKs indicates that the majority of them are associated with plant development or defense response, but there are also RLKs involved in cell wall attachment (extensin, proline-rich extensin and lectin RLKs), plant-bacterial symbiotic interactions (LysM RLKs) and self-incompatibility (S-domain containing RLKs). Among all RLKs, those bearing a leucine-rich repeat (LRR) domains are overrepresented in the RLK superfamily, comprising over 38% of Arabidopsis RLKs, which are distributed into 15 subgroups (LRR I to LRR XV). The LRR domains in these receptors vary in number (from one to 25) and in the distribution pattern of the LRRs along the extracellular region. Examples of well-known LRR-RLKs include CLAVATA1, which controls the size of stem cells in the apical meristem by forming a heterodimer with CLAVATA2 and then interacting with CLAVATA3 through the extracellular domain [9], and BRASSINOSTEROID INSENSITIVE-1 (BRI1) [10,11], which perceives brassinosteroids and interacts with it receptor partner, BAK1 (BRI1-ASSOCIATED KINASE-1) [12,13]. Other functions associated with LRR-RLKs include morphogenesis [14-20], embryogenesis [21-24], pollen self-incompatibility [25] and responses to environmental signals [26]. In addition, some LRR-RLKs are known to function as regulators of defense response to bacterial pathogen [27-29], necrotrophic fungus [30] and viral infection [31,32].

Most of the characterized RLKs are from model plants such as Arabidopsis and *Medicago truncatula*, but significant efforts have been made to expand these studies to relevant field crops. Large-scale comparative analyses of Arabidopsis RLKs with rice [5,33,34] and soybean [35] RLKs identified over 1000 kinase proteins in rice and 600 in soybean belonging to the RLK superfamily; almost all members were grouped into previously determined Arabidopsis RLK subfamilies. The RLK subfamilies with developmental function have conserved size, whereas those involved in defense response have expanded their members, mainly by tandem duplication [5].

Although tomato (*Solanum lycopersicum*) is one of the most consumed and cultivated field crops in the world, a large-scale phylogenetic analysis of tomato RLKs has not yet been performed, and few members of the tomato RLK/Pelle family (RLKs + RLCKs) have been studied and characterized. These members include Pto [36], Pti1 (Pto-INTERACTING 1) [37], and Bti9 (AvrPtoB-TOMATO INTERACTING PROTEIN 9) [38], which interact with *Pseudomonas syringae* elicitors; TARK1 (TOMATO ATYPICAL RECEPTOR KINASE-1) [39], which interacts with the *Xanthomonas campestris* elicitor; TPK1b (TOMATO PROTEIN KINASE 1) [40], whose expression is induced by mechanical wounding and oxidative stress; and SR160 (SYSTEMIN RECEPTOR) [41], which is the AtBRI1 ortholog and binds to systemin to respond to wounding or herbivore attack, although there is some debate about the function of this receptor [42]. Another well-studied RLK in tomato is NIK (NSP-INTERACTING KINASE), which interacts with nuclear shuttle protein (NSP) of geminivirus during infection [43]. Three homologs of NIK in Arabidopsis (AtNIK1, AtNIK2 and AtNIK3) have also been shown to interact with NSP through their kinase domain [31]. This interaction causes inhibition of the kinase activity of NIKs and hence prevents the activation of the signal transduction cascade that evokes a plant defense response [32]. These RLKs are members of the LRRII subfamily that also contains the SOMATIC EMBRYOGENESIS RECEPTOR KINASEs (SERKs) [44].

With the completion of the tomato genome sequencing along with the annotation of the encoded proteins [45], it has become possible to study the RLK superfamily in this species using a large-scale phylogenetic approach. According to genomic analyses, the tomato genome was predicted to have approximately 900 megabases of DNA and encode 34,727 proteins. In this investigation, we identified and classified all putative tomato RLKs by comparison with previously described Arabidopsis RLKs [5]. We also showed that the tomato RLK members of LRRII subfamily, which comprises *NIK* and *SERK* genes, share similar biochemical activity (capacity to interact with the geminivirus NSP), genomic structure and partial overlapping expression profiles with the Arabidopsis orthologs. Our results provide a framework for understanding RLK function in tomato and reveal that some tomato and Arabidopsis LRRII-RLK orthologs may play similar roles in antiviral defense.

## Results

### The tomato RLK superfamily

**Figure 1:**
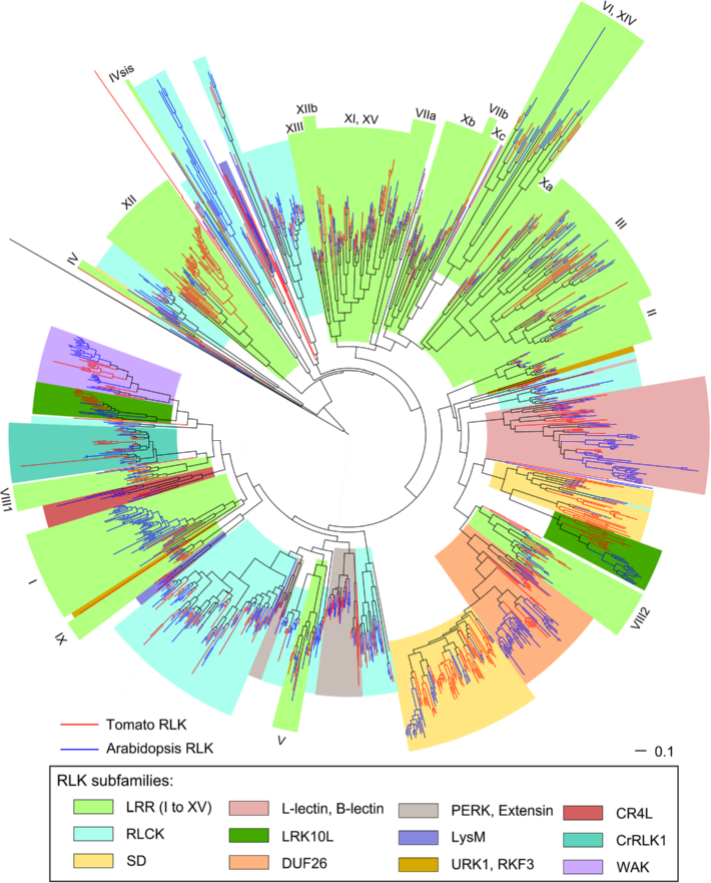
**The tomato RLK superfamily is composed of 647 proteins.** Phylogenetic tree constructed by sequence alignment of kinase domain of Arabidopsis RLKs together with putative tomato RLKs. The alignment was carried out with CLUSTALW, and the phylogenetic tree reconstruction was made using FastTree. Almost all tomato RLKs (red branches) clustered with Arabidopsis RLKs (blue branches). Color ranges delimit the RLK subfamilies. LRR subfamilies (light green) are subdivided in 15 groups, and each group is identified in the tree with Roman numerals (I to XV).

The identification of the RLK superfamily members in tomato was initially performed by a batch BLAST search against a tomato protein database (ITAG v2.3, available in solgenomics.net) using the kinase sequences of representative Arabidopsis RLKs as queries. This analysis retrieved 955 tomato proteins that seemed to be RLKs. All of these retrieved tomato proteins were submitted for annotation of their domain structure using SMART [46] (smart.embl-heidelberg.de) and Pfam [47] (pfam.sanger.ac.uk) databases. Four proteins that did not bear a kinase domain were not considered for further analysis. The remaining 951 proteins were used for phylogenetic analysis based on their kinase domain sequences. For this analysis, we included all Arabidopsis RLKs to compare with tomato RLKs and used representative proteins of other kinase families of Arabidopsis and human as outgroups (Additional file 1). All Arabidopsis RLKs were placed in a major cluster together with 647 tomato proteins that were identified as members of the RLK superfamily (Figure 1). The other 304 proteins were clustered with outgroups; consequently, they were not considered to be members of RLK superfamily (Additional file 2).

**Figure 2:**
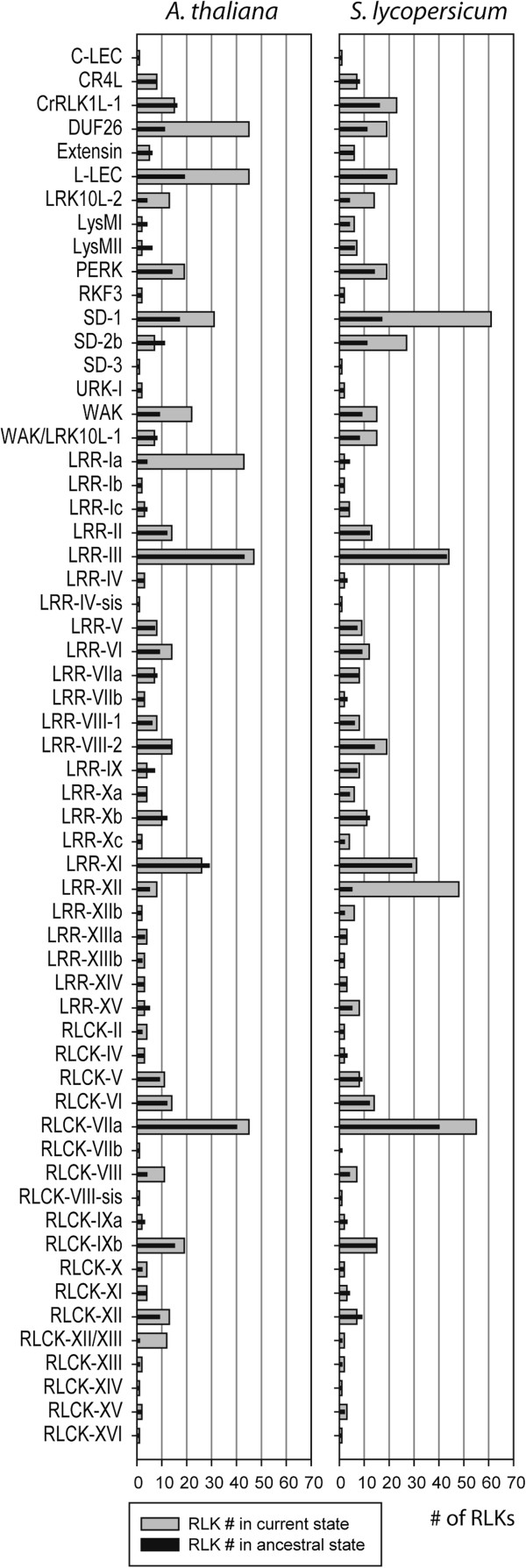
**The number of members varies in some tomato and Arabidopsis RLK subfamilies.** The distribution profile of tomato and Arabidopsis RLKs in subfamilies (gray bar) and the estimated number of RLK in their common ancestral (black bar) are presented. Almost all RLK subfamilies described in Arabidopsis have representatives in tomato.

The size of the tomato RLK superfamily (647 RLKs) was similar to that of the Arabidopsis RLK superfamily (623 RLKs). Furthermore, almost all tomato RLKs (631 RLKs) were clustered with at least one Arabidopsis RLK. Therefore, the tomato RLK superfamily was divided into the same 58 subfamilies as described previously for Arabidopsis [5]. As in Arabidopsis, in which 236 out of all 623 RLKs belong to leucine-rich repeat (LRR) subfamilies, tomato LRR subfamilies were the most abundant and contained 257 proteins. Another large RLK subfamily was RLCK, which included 128 members in tomato, almost the same number as in Arabidopsis (150). Among the 16 tomato RLKs that were not clustered in the same branches as Arabidopsis RLKs, ten proteins were quite small and lacked a typical RLK structure, but the other six proteins had a clear RLK structure and as such were considered to be tomato-specific RLKs. Among those six tomato-specific RLKs, Solyc03g080060 contained a legume lectin domain similar to members of the lectin subfamily, and Solyc02g083410 harbored an amino oxidase domain (flavin containing amine oxidoreductase activity), which is not found in any Arabidopsis RLKs. The remaining four proteins did not have any predicted protein domains in their extracellular region. RLK superfamily profiles in both species are summarized in Figure 2.

Although the RLK superfamilies of Arabidopsis and tomato share common features, a close inspection reveals some interesting differences between them. A comparison of membership size of each subfamily revealed some differences between the species. To infer the number of RLKs in their common ancestral and the occurrence of duplication/deletion events after the divergence of both species, we used a reconciliation method [48] to compare the RLK superfamily tree (Figure 1 and Additional file 2) with a species tree generated at NCBI taxonomy browser (Additional file 3). Then, the frequency of duplication or deletion events that occurred in each RLK subfamily was statistically analyzed. We identified twelve RLK subfamilies from these plant species that have significantly expanded/reduced in their size (Additional files 3 and 4, test of equal or given proportion, p< 0.05). In tomato, the LRK10L2, SD1, SD2b and LRRXII subfamilies displayed significant number of duplication, while in Arabidopsis, expansion was observed in DUF26, L-LEC, LRK10L2, SD1, WAK, LRRIa and RLCKXII/XIII subfamilies. Conversely, LysMII in Arabidopsis and LRRIa and RLCKXII in tomato reduced in size. Another distinct aspect of RLKs in these plant species refers to the lack of a common domain structure in the extracellular region of RLK members of the LRK10L-2 subfamily. Whereas the extracellular region of Arabidopsis LRK10L-2 members harbors diverse structures, such as thaumatin, glycerophosphoryl diester phosphodiesterase family (GDPD) or malectin domains, the tomato RLKs of this same subfamily do not contain any predicted domain structure in their N-terminal region.

Further analyses were performed to predict function associated with expansion/reduction patterns. As RLKs are frequently associated to defense or developmental processes, we performed a search using the Gene Ontology (GO) terms [49] for functionally annotated RLKs in those categories (Additional file 3) and statistically compared the proportion of annotated genes in each RLK subfamily with the proportion of annotated genes in the whole RLK superfamily (see Methods for more details). Compelling evidence in the literature has demonstrated that defense-related genes have high duplication rate and are organized in tandem repeats [34,50,51]. We also identified the RLKs organized in tandem repeats and determined their frequency in each RLK subfamilies (Additional file 3). These analyses demonstrated that most of the RLK subfamilies, which expanded after Arabidopsis and tomato species divergence, had their genes organized in tandem repeats. Functional annotation analysis of their genes revealed that the GO terms of 42 out of 214 genes in tandem repeats were associated with defense response. RLKs annotated as developmental-related were overrepresented in CrRLK1-1, PERK, LRRVIIa, LRRXI and LRRXIII subfamilies and all those subfamilies did not expand or present genes in tandem arrays. The subfamilies that underwent reduction in their size were not associated with defense- or development-related functions, except for the LRRIa subfamily, which may be related to defense response. Consistent with the involvement of members of the LRRII subfamily in defense and development, the LRRII subfamily of tomato and Arabidopsis had significantly high number of annotated members in either defense or developmental categories.

### Motif prediction, genomic structure and phylogenetic analysis of the LRRII subfamily

**Figure 3:**
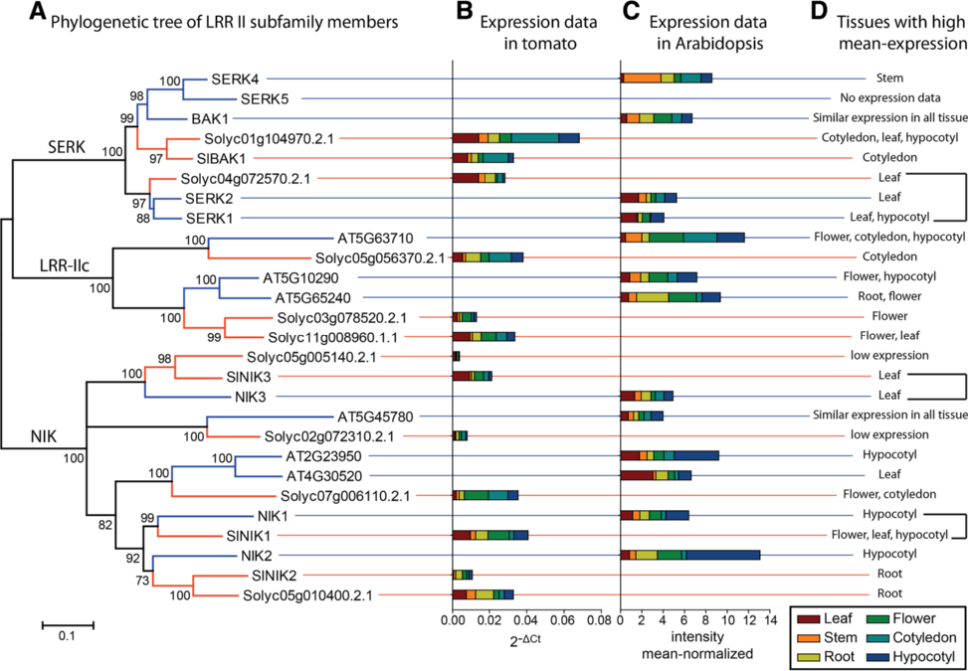
**Phylogenetic and expression analysis of LRRII subfamily members.** (**A**) Phylogenetic tree reconstructed by the maximum likelihood method (JTT+G+I, bootstrap replicates = 1000) of the LRRII subfamily. Members of this subfamily can be separated in three well-supported clades, referred to here as SERK, NIK and LRRIIc clades. Expression analysis of LRRII subfamily members in (**B**) tomato and (**C**) Arabidopsis in different plant tissues. The expression data of tomato and Arabidopsis members were obtained by qRT-PCR and from normalized data from the AtGenExpress database [55], respectively. No expression data were obtained for AtSERK5 (At2g13800). (**D**) Tissues with high mean-expression are summarized for each gene. Orthologous genes that had similar expression profiles are delimited by brackets.

**Figure 4:**
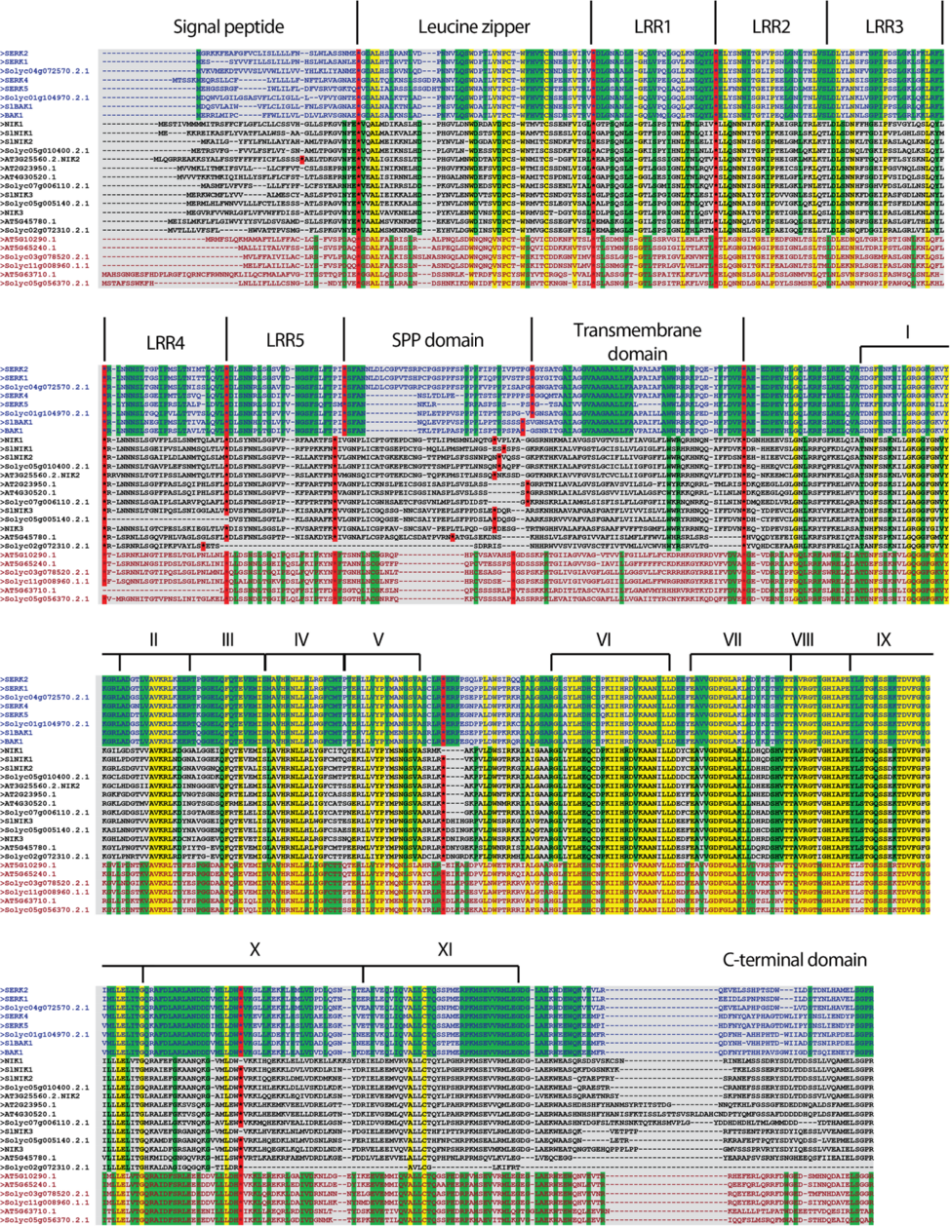
**Full-length sequence alignment of LRRII subfamily members of tomato and Arabidopsis demonstrate sequence and structure conservation.** Sequences of SERK, NIK and LRRIIc clades members are represented with blue, black and red letters, respectively. Yellow sites represent conserved sites in all sequences, and green sites represent conserved sites in each clade. Red sites represent the exon-exon junctions. Domain structures are indicated above the alignment. Roman numerals delimit the 11 subdomains of the kinase domain.

**Figure 5:**
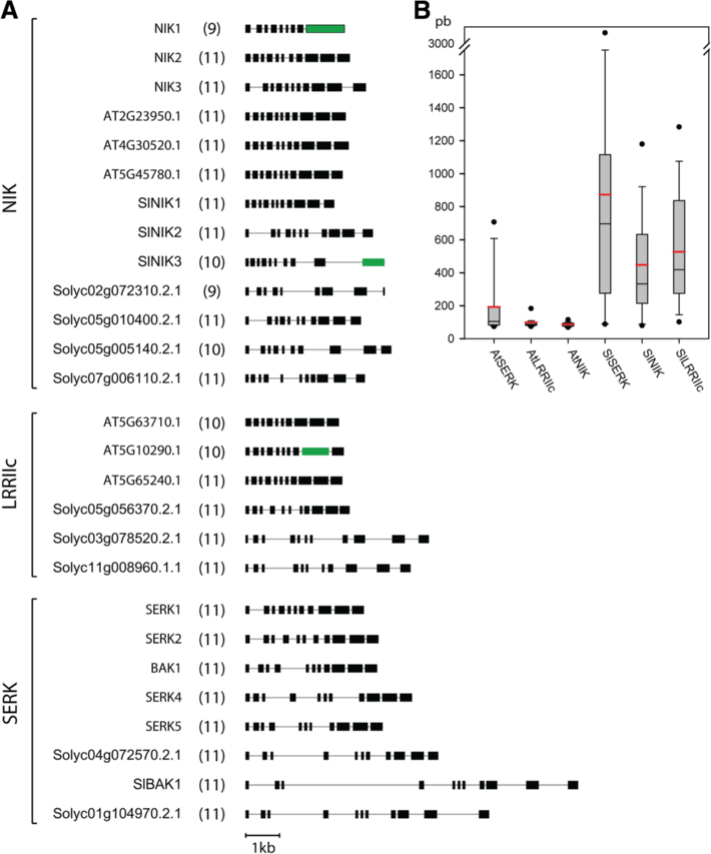
**Genomic structure analysis of members of the LRRII subfamily of tomato and Arabidopsis.** (**A**) Exons are shown as dark boxes and introns as grey lines. Green boxes represent fused exons. The number between parentheses represents the number of exons in each gene. Almost all genes contain 11 exons. (**B**) Boxplot illustrating the distribution of intron length among LRRII clades of Arabidopsis and tomato. The red line marks the average intron length. Note the large length of intronic regions in SERK genes compared with genes from other clades, and in tomato sequences compared with Arabidopsis sequences.

Compelling evidence in the literature has revealed a fundamental role for members of the Arabidopsis LRRII-RLK subfamily as co-receptors for transducing developmental and defense signals [52-54]. The potential of the members of this subfamily as co-receptors involved in the activation of RLK-mediated signal transduction prompted us to perform a comprehensive analysis of the tomato LRRII-RLK subfamily to uncover related functions in tomatoes. Based on the phylogenetic tree of all members of RLK superfamily, the tomato LRRII-RLK subfamily encompassed 13 proteins. The members of this group from both plant species have over 600 amino acids on average. Phylogenetic analysis of this group using full-length protein sequences resulted in a tree with three well-resolved clusters; the tomato and Arabidopsis proteins were found in all clusters, although they had distinct sizes (Figure 3A). These clades were termed NIK, SERK and LRRIIc based on annotation of the Arabidopsis members in each cluster. The NIK clade is formed by seven tomato members and six Arabidopsis members, including the three AtNIK genes. The SERK clade clustered the five well-characterized SERKs in Arabidopsis and three members of the tomato subfamily. The LRRIIc clade consisted of three tomato proteins and three Arabidopsis proteins whose functions are unknown. Motif prediction analysis on these proteins revealed that tomato and Arabidopsis LRRII-RLK members display similar protein domains organized in the same fashion (Figure 4). The consensus structural organization of the conserved domains between both species included an N-terminal signal peptide followed by a leucine zipper, five LRRs at the extracellular side and a transmembrane domain separating the N-terminal portion from the cytoplasmic C-terminal kinase domain. Among the *SERK* genes of both species, there was also a proline-rich domain (SPP) localized between the last LRR and the transmembrane domain (Figure 4). Sequence alignment of the LRRII RLKs showed several conserved amino acid positions among members of both plant species. Exon/intron boundaries were also well conserved. Variation at the sequence level was observed within the SPP and signal peptide recognition domains. Among the LRRII clades, the proteins comprising the SERK clade were more conserved with a larger number of conserved positions compared with the predicted proteins of the LRRIIc and NIK clades (Figure 4). Genomic structure analysis revealed that in general LRRII genes are organized into 11 exons (Figure 5A). Genes that varied in this number displayed fused exons, including *AtNIK1*, At5g10290.1 and *SlNIK3*, or had deleted exons, such as At5g63710.1, Solyc02g072310.2.1 and Solyc05g005140.2.1. Intronic regions were larger in tomato members than in Arabidopsis members and in *SERK* genes compared with genes from other clades (Figure 5B).

### Expression analysis of LRRII subfamily genes in different tissues

**Figure 6:**
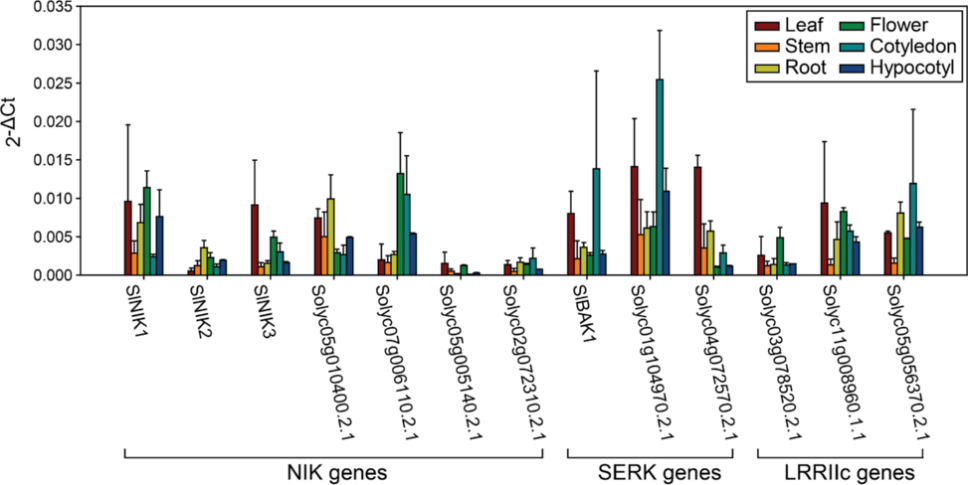
**Expression analyses of tomato members of the LRRII subfamily in various plant organs by qRT-PCR.** Expression of each gene was quantified using *SlAPT1* as an endogenous control. Bars represent the mean expression from three biological samples and two replicates, except for the flower and hypocotyl samples, for which two biological samples and two replicates were used. Error bars represent a confidence interval of 95%.

We examined the expression profiles of LRRII-RLK genes in different tomato tissues, including leaf, stem, root, flower, cotyledon and hypocotyl, by real-time PCR. The results are presented in Figure 6 and summarized in Figure 3B. Almost all analyzed genes exhibited expression in at least one organ, except for Solyc05g005140.2.1 and Solyc02g072310.2.1 in the NIK clade, which had very low expression in all tissues tested. The tomato genes in the SERK clade were expressed more highly in leaf and cotyledon tissue than in stem or flower tissue. The NIK clade genes were highly expressed in diverse organs, such as leaves, flowers and roots. The LRRIIc group encompassed genes with a higher level of expression in cotyledon, flower and leaf tissues and with lower expression in stem tissue.

The expression data for LRRII subfamily members from Arabidopsis (Figure 3C) were extracted from AtGenExpress [55] to examine whether there is some correspondence in the expression profiles between orthologous pairs of tomato and Arabidopsis genes. Statistical analyses of correlation between tomato and Arabidopsis expression data could not be performed because these data have been generated by different methods (qRT-PCR and microarray) and hence they have different units. Nevertheless, a subjective comparison of the expression analysis from both plants revealed that the majority of the orthologous genes displayed partially but not entirely overlapping expression profiles (Figure 3D). The orthologous groups that presented similar expression profile were *AtSERK1*/*AtSERK2* and Solyc04g072570, which had high expression levels in leaves, *AtNIK1* and *SlNIK1*, which were lowly expressed in stem and cotyledon tissues, and *AtNIK3* and *SlNIK3*, which were most highly expressed in the leaf.

### Interactions between representatives of the LRRII subfamily and NSP of ToYSV

**Figure 7:**
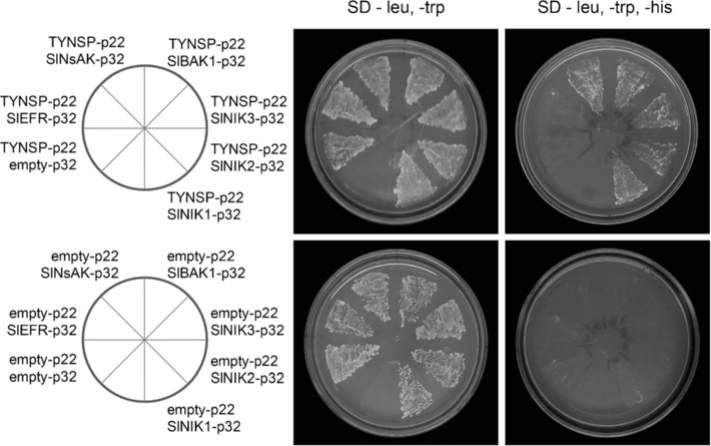
**Tomato members of the LRRII and PERK subfamilies interact with NSP of ToYSV.** Yeast two-hybrid assay using the kinase domain of LRRII and PERK subfamily members of tomato as bait and the NSP of ToYSV as prey. All co-transformed yeast strains were grown on synthetic defined (SD) medium lacking leucine and tryptophan (SD-leu, -trp), indicating the presence of both plasmids constructs in their cells. Yeast growth on SD medium lacking leucine, tryptophan and histidine (SD -leu, -trp, -his) indicates an interaction between the bait and prey constructs. This was observed in the yeast strains co-expressing NSP and SlNIK1, SlNIK2, SlNIK3, SlBAK1 or SlNsAK. No interaction between NSP and SlEFR was observed. All negative controls using empty vector failed to grow on SD -leu, -trp, -his, indicating the absence of transactivation.

We have previously shown that NSP from begomovirus interacts with members of the LRRII-RLK subfamily, such as AtNIK1, AtNIK2 and AtNIK3, to suppress host defense, and it interacts with a member of the PERK-like RLK subfamily, NSP-ASSOCIATED KINASE (NsAK), to potentiate virus infection [31,56]. Either NIK from tomato and NsAK from Arabidopsis were isolated by their capacity to interact with NSP through two-hybrid screening [43,56]. The NSP interactions with the Arabidopsis AtNIK1, AtNIK2 and AtNIK3 and NsAK were further confirmed by yeast-two hybrid and in vitro pull down assays [31,56]. We have recently shown by bimolecular fluorescence complementation (BiFC) assay that NSP also interacts with NIK *in vivo*. Because begomovirus negatively impacts tomato cultivation worldwide, we selected representatives of tomato RLKs from the LRRII subfamily and examined their capability to interact with NSP of *Tomato Yellow Spot Virus* (ToYSV) similar to the interaction observed with Arabidopsis *NIK* genes [31]. Yeast two-hybrid experiments were performed using the ToYSV-NSP (accession number: YP_459917.1) as prey and kinase domains of SlNIK1 (Solyc02g089550), SlNIK2 (Solyc04g005910), SlNIK3 (Solyc04g039730) and SlBAK1 (Solyc10g047140) as bait. We also analyzed a PERK representative (Solyc12g007110, SlNsAK) that is similar to the NSP-interactor PERK-like gene of Arabidopsis (At5g24550, AtNsAK). A tomato gene (Solyc03g019980) from the LRRXII subfamily, homolog of the Arabidopsis EF-Tu receptor (AtEFR), was used as a negative control. Interactions between the viral NSP and host proteins were detected after co-transforming the yeast cells with both bait and prey plasmids and monitoring for histidine prototrophy. NSP was found to interact with the kinase domains of SlNIK1, SlNIK2, SlNIK3 and SlBAK1 or with the kinase domain of the PERK representative SlNsAK (Figure 7, upper panel). The NSP interactions were specific to the tomato LRRII-RLK orthologs and to PERK-like SlNsAK because the *HIS* marker gene was not activated in yeast cells co-transformed with TYNSP-p22 (pAD-NSP) and with either the empty vector or SlEFR-p32 (EFR kinase domain). Furthermore, co-transformation of yeast with the NSP interactors fused to the GAL4-binding domain and the empty vector expressing the GAL4-activating domain alone also failed to activate the *HIS* marker gene (Figure 7, lower panel). These results expanded our previous observation that NSP-NIK complex formation was neither virus-specific nor host-specific [31,43]. They also suggest that SlNsAK is a NSP target during begomovirus infection in tomato. Certainly, the *in vivo* demonstration of these interactions will further support these interpretations.

## Discussion

### The structure of the tomato RLK superfamily and the proposed evolution of RLK superfamily in plants

**Figure 8:**
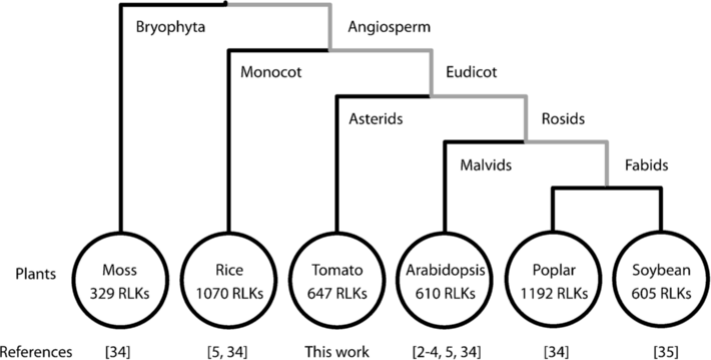
**Cladogram of plants whose RLK superfamily has been characterized.** The RLK superfamily size ranges from 329 (moss) to 1192 (poplar) members. The RLK superfamily expanded after divergence between the Bryophyta and Angiosperm lineages and independently expanded in the plants of the Angiosperm lineage. Dramatic expansions were observed in rice and poplar.

To date, the phylogenetic and structural characterization of the RLK superfamily has been limited to the following plant species: moss, rice, poplar, soybean and Arabidopsis [5,34,35]. The size of these families ranges from 300 to 1200 proteins (Figure 8), and their extracellular regions bear a great variety of protein domain structures. In the present investigation, we characterized and generated a complete list of the tomato RLK superfamily members (Additional files 2 and 5), identifying 647 RLKs, which falls in the size range of the Arabidopsis (623 RLKs) and soybean (605 RLKs) superfamilies.

Evolutionary analyses of the RLK superfamily has suggested that the RLK structure was established prior to the divergence of land plants from algae because proteins with RLK configurations were discovered in the unicellular algae *Chlamydomonas reinhardtii*[34]. Comparative analysis of RLKs among moss, rice, poplar and Arabidopsis revealed that the RLK superfamily underwent expansion in the beginning of the land plant lineage, after the divergence of angiosperm and bryophyte and independently during diversification of each angiosperm lineage. The most dramatic expansion was observed in the rice and poplar lineages, which have almost twice as many as RLK members as Arabidopsis [34]. This evolutionary scenario has not been changed by inclusion of data regarding the soybean [35] and tomato (this work) RLK superfamily expansion (Figure 8).

The phylogenetic tree of the members of the RLK superfamilies in tomato and Arabidopsis revealed that most of the RLK subfamilies have maintained approximately the same number of RLK members between these species. Exceptions were observed for the DUF26, L-LEC, LRK10L2, SD1, SD2b, WAK, LRRIa, LRRXII, LRRXIIb, RLCKXII/XIII subfamilies, in which specific and extensive expansion was observed in one of the two plant species, as well as for the LysMII, LRRIa and RLCKXII, in which specific reduction was observed (Additional file 3). Functional annotations of some Arabidopsis RLKs and the number of genes in tandem repeat that compose those subfamilies indicated a predominance of genes clustered in tandem array and defense-related RLKs (Additional file 3). Some of those subfamilies, such as DUF26, L-LEC, SD1, SD2b, WAK and LRRIa, had been shown previously to be overrepresented in microarray analysis of Arabidopsis under different stress conditions [34]. Taken together, these results are consistent with the previous assumption that specific expansions of RLK genes have occurred more frequently for those RLKs associated with defense response.

Specific expansion of tomato RLKs compared to Arabidopsis occurred in the LRK10L2, LRRXII, SD1 and SD2b subfamilies. Interestingly, all of those RLK subfamilies, except for SD1, were also overrepresented in rice when compared with Arabidopsis [5]. The LRRXII subfamily comprises the EF-Tu RECEPTOR (EFR) and FLAGELLIN-SENSITIVE 2 (FLS2) in Arabidopsis, and Xa21 in rice, all of which are associated with defense responses. The expansion of the LRRXII subfamily in cultivated plants such as tomato and rice has previously been suggested to be associated with the accumulation of resistance genes by intense breeding programs [5]. Likewise, the SD subfamily also has representatives involved in defense response, such as RK1 and RK3 [57], but this subfamily is also strongly associated with the self-incompatibility process [58]. Arabidopsis is known to be self-compatible, while rice, tomato and the close relative of *A. thaliana*, *A. lyrata*, are self-incompatible. Thus, the specific deletion of some representatives in *A. thaliana* could have contributed to the generation of the self-compatible mode in this species. In contrast, some RLK subfamilies were expanded specifically in Arabidopsis. Those include DUF26, L-LEC, LRK10L2, SD1, WAK, LRRIa and RLCKXII/XIII. Except for SD1 and LRK10L2 subfamilies, no significant expansion were observed in these RLK subfamilies in tomato, although all of them contain RLKs that are involved in defense response, such as FLS2-INDUCED RECEPTOR-KINASE 1 (FRK1, LRRIa) [59], LECTIN RECEPTOR KINASE 1.9 (LecRK-1.9, L-LEC) [60], PATHOGENESIS-RELATED 5-LIKE RECEPTOR KINASE (PR5K, LRK10L2) [61], RESISTANT TO FUSARIUM OXYSPORIUM 1 (RFO1, WAK) [62] and CYSTEINE-RICH RLK 5 (CRK5, DUF26) [63].

Another relevant distinction between tomato and Arabidopsis RLK subfamilies derives from the diversification of the extracellular domain patterns of the LRK10L2 subfamily representatives. Arabidopsis members of the LRK10L2 subfamily have unique domain structures, such as GDPD, thaumatin and malectin domains, while the tomato members do not possess any characterized domain structure in their extracellular region. Likewise, these domain structures have not been found in rice, poplar or moss RLKs, indicating that within the RLK superfamily they are specific to Arabidopsis. We also identified a tomato-specific RLK that possesses an amino oxidase domain in its extracellular region. These RLKs may respond to molecular signals not perceived by other plants. Although gaining a novel protein structure could increase the repertoire of signals perceived by plants, the small number of lineage-specific RLKs in tomato, as was also reported in rice and poplar, further substantiates the hypothesis that the expansion of existing RLK kinase subfamilies is the major mechanism of evolution of these proteins.

The members of the RLK superfamily are involved in diverse biological processes at all steps of plant development. Thus, the gain or loss of a RLK gene could have serious repercussions on plant phenotype. The specific profiles of the RLK superfamily found in tomato and Arabidopsis are certainly responsible for several differences between these plants, such as morphology, reproduction and, importantly, responsiveness to different stress conditions. Among tomato and Arabidopsis, few RLK subfamilies have undergone specific expansion or reduction after their speciation. This scarcity may indicate that variation in RLK superfamily profiles in both plants appeared recently. The domestication process that tomatoes underwent could have been a significant factor contributing to this variation because some RLKs have been directly linked to traits targeted by artificial selection, such as disease resistance and growth. Nevertheless, most of the specific expansions of RLK subfamilies observed in Arabidopsis were also associated with defense response and had occurred at similar rates (287 and 259 duplication events in Arabidopsis and tomato, respectively). These data suggest that, independently of artificial selection, Arabidopsis had also expanded and developed a specific machinery against abiotic or biotic stress response, which argues against the assumption that artificial selection leads to resistant genes accumulation. To further examine the influence of artificial selection on the repertoire of plant RLKs, new genetic resources for closely related wild plants are necessary.

### Functional expression analysis of the LRR II subfamily members in tomato and Arabidopsis

The LRRII subfamily contains RLKs with dual functions in development and defense response [52-54]. Characterized members of this subfamily include (i) *SERK* genes, which are associated with diverse processes, such as brassinosteroid signaling, flagellin, cell death, light and pathogen-associated molecular pattern (PAMP) responses [53], and (ii) *NIK* genes, which interact directly with geminivirus NSP during viral infection [31,32]. Phylogenetic and protein structure analyses on LRRII subfamily members of tomato and Arabidopsis demonstrated that this group is highly conserved between these species. In rice, in which the RLK superfamily has undergone a large expansion, the LRRII subfamily members are also conserved in number and sequence, indicating that biochemical pathways regulated by LRRII-RLKs have essential and conserved roles in angiosperm species.

Although LRRII members have well-conserved amino acid sequences among various species [64], expression analysis of the members of the tomato and Arabidopsis LRRII subfamilies demonstrated that only a few of the orthologous pairs resemble in their expression profiles. By analogy with some evidence in the literature from other plant species, one may envision that these orthologous genes could have functionally diverged after the speciation event separating tomato and Arabidopsis. Functional divergence in orthologous genes is not an uncommon event in both plants [65,66] and animals [67,68]. For example, the CRABS CLAW transcription factor in Arabidopsis is expressed in the carpel primordial abaxial region and in floral nectarines and regulates carpel morphology and nectar development, whereas its orthologous in rice, DROOPING LEAF (DL), is expressed in the whole carpel primordium and in central undifferentiated cells of leaves, where it regulates carpel identity and midrib development [66]. The expression of orthologous genes has also been shown to vary differently in response to a stress condition. In barley, which is tolerant to salinity, the expression of genes involved in root development, such as CONSTANS-LIKE 3 (COL3), is suppressed by high salinity, whereas the expression of the rice orthologous is unchanged under the same stress condition [65]. Likewise, a large fraction of orthologous pairs of rice and Arabidopsis genes with receptor activity do not display conserved co-expression [69]. Therefore, different patterns of expression between the orthologous genes in the LRRII subfamilies from tomato and Arabidopsis may be a result of functional divergence that occurred between these genes. Functional divergence in receptor proteins with a developmental function may lead to a dramatic change in the plant phenotype because plant development is heavily guided by external signals. For example, a tissue that displays high expression of certain RLKs is likely to be more sensitive to perception of RLK-specific sensing signals, leading to a rapid and effective response. In contrast, reduced expression of an orthologous gene from a different species in the same tissue would decrease the effectiveness and delay the signal perception and response. This difference in the cell responsiveness to a specific signal could represent the differential timing of biochemical reactions that are regulated by this signal. In developmental process, small differences in reaction time may be sufficient to generate a distinct phenotype in the plant. In contrast, the orthologous pairs SERK1/SERK2/Solyc04g072570.2.1, which display similar expression profiles (Figure 3), also contain the most conserved extracellular and intracellular domains (approximately 80% and 93% of sequence identity, respectively). The other two orthologous pairs, NIK1/SlNIK1 and NIK3/SlNIK3, which also displayed similar expression profiles, also had highly conserved extracellular and intracellular regions. In both orthologous pairs, sequence identity was approximately 65% in the extracellular regions and approximately 80% in the intracellular regions (Additional file 6). This finding may indicate a tight conservation of function between these members of the Arabidopsis and tomato LRRII RLK subfamilies.

### Conservation of geminivirus interactions with members of the RLK family in tomato

Although most of LRRII subfamily orthologous pairs exhibited functional divergence, we showed that the tomato orthologs of the LRRII-RLKs members NIK1, NIK2 and NIK3 retain the capacity to interact with geminivirus NSP in yeast (Figure 7) [31]. At least for the NIK1 and NIK3 ortholog pairs, the functional conservation associated with specific protein-protein interactions may be linked to the high conservation of their NSP-interacting kinase domain (approximately 80% sequence identity, Additional file 6) and similarity of expression profiles (Figure 3). The current model of NIK-mediated defense response posits that the immune receptor protects plant against geminiviruses by phosphorylating the ribosomal protein L10 (rpL10) [32,70]. Phosphorylation of rpL10 by NIK redirects the ribosomal protein to the nucleus, where it may mount a defense mechanism to prevent viral proliferation. During geminivirus infection, NSP interacts with the kinase domain of NIKs to inhibit their kinase activity, preventing activation of the defense response. Despite the high similarity between *NIK* genes and *SERK* genes, AtBAK1/SERK3 and AtSERK1 do not functionally replace the AtNIK1 role in transducing an antiviral signaling response and do not interact with the viral NSP [31,32]. In contrast, we found that the *AtBAK1* ortholog from tomato interacts with NSP in yeast. Although the functional relevance of this interaction *in planta* remains to be determined, it is worth noting that the expression profiles of the *BAK1* orthologs are not similar, as would be expected for functionally divergent orthologs. Although both orthologs are ubiquitously expressed in the cognate plant species, they are expressed to different extents in distinct organs. Whereas AtBAK1 expression is quantitatively similar and relatively low in all organs analyzed, its ortholog from tomato displays a higher level of expression in the cotyledons, hypocotyls and leaves, where geminivirus infection largely takes place. Therefore, the expression profiles of the NSP interactors (SlNIKs and SlBAK1) seemed to be related with the onset of geminivirus infection. Due to the high expression of the AtBAK1 tomato orthologous in leaves, one may envision the existence of evolving selective pressures to diverge the corresponding NSP-interacting domains of the BAK1 orthologs towards functional fitness with regard to geminivirus infection.

In contrast to NIK receptors, which are inhibited by NSP interaction, AtNsAK, a member of PERK subfamily, interacts with NSP and phosphorylates the viral protein *in vitro*[56]. Loss of *nsak* function enhances tolerance to geminivirus infection, indicating that AtNsAK is a positive contributor to geminivirus infection in Arabidopsis. Here, we showed that the NsAK tomato orthologous retains its capacity to interact with viral NSP. This demonstrates that specific members of the RLK family have conserved defense functions (such as NIKs) or compatibility functions (such as NsAK) in response to viral infection. Due to the emergence of new species of tomato-infecting begomoviruses that rapidly evolve through recombination or pseudo-recombination to produce divergent genome sequences that gives the virus an advantage over its host’s recognition system, a survey of the interactions between NSPs from distinct tomato-infecting geminiviruses and SlNIKs and SlNsAK may add insights into the co-evolution of the viral protein and host defense/compatibility functions.

## Conclusions

The RLK superfamily is a large and diverse group of transmembrane receptors that enables plants to perceive a diverse array of signals at the cell surface, creating an efficient mechanism for cell-environment communication. In this investigation, we generated a complete list of the members of the tomato RLK superfamily, which is made-up of 647 proteins. The tomato RLK sequences exhibited a typical receptor-like kinase configuration and almost all of them were phylogenetically clustered with at least one member of the Arabidopsis RLK superfamily. Therefore, the tomato RLK superfamily is similarly organized, with the same number and identity of subfamilies as previously defined for Arabidopsis RLKs. Among the 58 RLK subfamilies, twelve showed specific and extensive expansion or reduction in the number of their RLK members, which may be a reflection of lineage-specific responses to various biotic and abiotic stresses. The intense breeding programs tomatoes have been subjected to may also have contributed to the establishment of the current RLK superfamily profile in this species. This comprehensive analysis comparing the complete repertory of Arabidopsis and tomato RLKs may provide a framework to rationalize future functional studies of the members of this family.

Phylogenetic and structural analyses of LRRII subfamily members from both tomato and Arabidopsis reveal a well-conserved group both in terms of sequence and protein domain organization. As a consequence, the tomato LRRII-RLK subfamily is organized into the same three with phylogenetically supported clades, SERK, NIK and LRRIIc clusters. Nevertheless, a comparison of the expression between orthologous genes of this subfamily demonstrated that the majority of the orthologous pairs did not share a similar expression profile, indicating that these orthologous LRRII-RLKs may have undergone functional divergence. This finding is supported by the observation that, in contrast to the Arabidopsis AtBAK1, SlBAK1 interacts with the geminivirus NSP and is highly expressed in leaves and the cotyledon. This pattern of SlBAK1 expression is consistent with the pattern of infection by tomato-infecting begomoviruses, which infect leaf tissues and move through the phloem but do not invade roots. Additionally, as immune receptors, the orthologous pairs NIK1 and NIK3 displayed both the capacity to interact with the begomovirus virulence factor NSP and expression profiles that parallel the onset of begomovirus infection. Evidence for functional conservation between NIK1 orthologs has been previously provided with the demonstration that NIK1 from Arabidopsis is capable of protecting tomato plants against tomato-infecting begomovirus [70]. Collectively, our results indicate that NIK orthologs retain similar functions as defense receptors to protect plant cells against viral attack. Therefore, NIK-mediated antiviral signaling likely also operates in tomato, suggesting that the tomato NIKs may be good candidate targets for engineering resistance against tomato-infecting begomoviruses.

## Methods

### Identification and classification of tomato RLKs

Tomato RLK proteins were retrieved through a batch BLAST analysis (blastp, e-value cutoff = 0.01) [71] using an *A. thaliana* representative of each subfamily of the RLK superfamily against a protein database of tomato (iTAGv2.3) available on the Sol Genomics Network website (solgenomics.net) [72]. Through this procedure, 955 predicted proteins were retrieved and annotated using SMART (smart.embl-heidelberg.de) [46] and Pfam (pfam.sanger.ac.uk) [47] databases. Among these proteins, 951 contained a predicted kinase domain and hence were considered to be putative RLKs. The sequences of the kinase domains of Arabidopsis RLKs, previously described in [5], and tomato putative RLKs were submitted to sequence alignment and tree reconstruction using ClustalW (v. 2.0.12) [73] and FastTree (v. 2.1.4) [74], respectively (Figure 1 and Additional file 2) using default parameters. The kinase domain of other kinase protein families from *A. thaliana* and human were used as outgroups [3,75]. The accession numbers for all outgroup members are reported in Additional file 1. Those proteins that clustered with outgroup members were not considered to be RLKs and were discarded from further analysis. Additionally, short putative RLKs were deleted manually from the analysis. The identified RLK-related tomato sequences comprised a list of 647 members. Tomato RLKs that clustered with *A. thaliana* RLK subfamily members, as defined previously in [5], were classified as members of the same subfamily. Phylogenetic trees (Figure 1 and Additional file 2) and protein schemes (Additional file 2) were generated using iTOL tool (itol.embl.de) [76].

### Inference on duplication/deletion events, identification of RLKs in tandem repeats and functional categorization of RLKs subfamilies

Number of members in the common ancestral of Arabidopsis and tomato and the occurrence of gene duplication and deletion were inferred by reconciliation methods implemented in Notung (v.2.6) [48]. For this analysis, we used the RLK superfamily tree, showed in Figure 1 and in Additional file 2, as gene tree. The species tree was generated at NCBI taxonomy browser ( http://www.ncbi.nlm.nih.gov/Taxonomy/CommonTree/wwwcmt.cgi). For identification of RLKs in tandem repeats, we considered that two genes are clustered in tandem array when i) they are classified in the same subfamily, ii) they are distant from each other by less than 100kb and iii) they are separated by less than 10 genes from each other, as previously described in [34]. For identification of defense- or development-related genes, we used the GO terms associated to the Arabidopsis genes. Arabidopsis RLKs that had GO terms related to "response to stress" (GO:0006950) and/or "developmental process" (GO:0032502) and their child terms were classified as defense- and/or developmental-related, respectively.

### Statistical test for expansion/reduction analysis and functional categorization of RLK subfamilies

To statistically verify if (i) RLK subfamilies have differentially expanded or reduced in their size, (ii) tandem duplications or (iii) a functional annotation (defense or development) are more often in a specific RLK subfamily, we used the test of equal or given proportions [77]. This statistical analysis tests if two different proportions (p_1_ and p_2_) are equal (H_0_:p_1_ = p_2_) or different (H_a_: p_1_ ≠ p_2_). The two tested proportions were the occurrence of a given feature (number of duplication/deletion, tandem repeats or genes annotated as defense- or developmental-related) in a subpopulation (RLK subfamily, p_1_), and the proportion of the number of the same feature in the whole population (RLK superfamily, p_2_). As we analyzed whether those features were overrepresented in a given RLK subfamily, our alternative hypothesis was H_a_: p1 > p2. Test calculations were performed in R environment. All p-values associated with tested values are summarized in Additional file 4.

### Motif prediction, genomic structure and phylogenetic analysis of the LRRII subfamily

Full-length amino acid sequences of members of the LRRII subfamily from tomato and Arabidopsis were aligned using ClustalW (v. 2.0.12) [73] using the default parameters. A phylogenetic tree was constructed using the maximum likelihood method (JTT model, bootstrap replicates = 1000) implemented in MEGA5 software [78]. Motif, signal peptide and transmembrane prediction were carried out using Pfam [47] and SMART [46] databases. The genomic structure of the LRRII subfamily members of tomato and Arabidopsis was determined by aligning the coding sequence (CDS) of each gene with genomic sequences of the respective organism. The alignment was carried out using the BLAST algorithm (blastn) [71] with high-stringency parameters. Amino acid, CDS and genomic sequences for tomato and Arabidopsis were retrieved from the Sol Genomics Network (solgenomics.net) [72] and TAIR ( http://www.arabidopsis.org) [79] websites, respectively.

### Protein-protein interaction assays

The analysis of protein-protein interactions between viral NSP and the kinase domain of tomato RLKs was performed using the Proquest Yeast Two-Hybrid system with Gateway Technology (Invitrogen Inc.). The tomato RLKs that presented the highest identity with AtNIK1 (At5g16000), AtNIK2 (At3g25560), AtNIK3 (At1g60800), AtBAK1 (At4g33430) and AtNsAK (At5g24550) were selected for the assay. These tomato proteins are referred to as SlNIK1 (Solyc02g089550), SlNIK2 (Solyc04g005910), SlNIK3 (Solyc04g039730), SlBAK1 (Solyc10g047140) and SlNsAK (Solyc12g007110). As a negative control, we used the kinase domain of the tomato RLK that displayed the highest identity with AtEFR (At5g20480), referred to as SlEFR (Solyc03g019980).

The NSP coding region was amplified from ToYSV (Tomato Yellow Spot Virus-Geminiviridae, *Begomovirus*) [80] using gene-specific primers with appropriate extensions for cloning via the Gateway system, as described in Additional file 7. The amplified fragment was cloned into pDONR201 to generate pUFV1780.1 and then transferred by recombination to pDEST22 yielding pUFV1781, also designated as TYNSP-p22.

For amplification of the C-terminal kinase domain of the tomato RLKs, we prepared cDNA from cotyledons of wild-type tomato plants (var. Santa Clara). Briefly, total RNA from tomato cotyledons was isolated using an RNeasy Kit (Qiagen Inc.). First-strand cDNA was synthesized from 1 μg of total RNA using the M-MLV Reverse Transcriptase (Invitrogen Inc.) according to the manufacturer’s instructions. Primers used in the amplification step were designed with recombination sites for further cloning procedures using the Gateway System (Invitrogen Inc.). The primers used are listed in Additional file 7. PCR assays were performed using Platinum *Taq* DNA Polymerase High Fidelity (Invitrogen Inc.) according to the manufacturer‘s instructions. The amplified fragments were cloned into the entry vector pDONR201 (Invitrogen Inc.) and sequenced. The resulting vectors were the following: pUFV1756.1, pUFV1596, pUFV1757.1, pUFV1734.2, pUFV1744.1 and pUFV1955.2, corresponding, respectively to the fragment encoding the kinase domain of SlNIK1, SlNIK2, SlNIK3, SlBAK1, SlNsAK and SlEFR. Then, the cloned fragment in pDONR201 was transferred to pDEST32, which contains the DNA-binding domain of the GAL4 promoter (Invitrogen Inc.). This procedure resulted in the following recombinant plasmids: pUFV1768.1, pUFV1760.1, pUFV1779.1, pUFV1769.1, pUFV1770.1 and pUFV1975.1, also designated as SlNIK1-p32, SlNIK2-p32, SlNIK3-p32, SlBAK1-p32, SlNsAK-p32 and SlEFR-p32, respectively.

Competent cells of yeast strain AH109 (Clontech Inc., genotype: *MATα*, *trp1-901*, *leu2-3,112*, *ura3-52*, *his3200*, *gal4Δ*, *gal80Δ*, *LYS2∷GAL1*_*UAS*_*-GAL1*_*TATA*_*-HIS3*, *GAL2*_*UAS*_*-GAL2*_*TATA*_*-ADE2*, *URA3∷MEL1*_*UAS*_*-MEL1*_*TATA*_*-lacZ*) were sequentially co-transformed with TYNSP-p22 and with one of the pDEST32 constructs. Co-transformed yeasts were plated onto synthetic dropout medium lacking leucine, tryptophan and histidine, and incubated at 28°C. Yeast growth was monitored for 5 days.

### Expression analysis of the LRRII subfamily genes

The expression patterns of genes in the LRRII subfamily were assayed by quantitative Real-Time PCR (qRT-PCR) in various tomato tissues. Wild-type tomato plants (var. Santa Clara) were cultivated in a greenhouse for 45 days after germination. Leafs, stems, roots and flowers from three plants were collected separately. We also cultivated plants in half-strength Murashige and Skoog medium (1/2 MS, Sigma-Aldrich Co.) for 10 days after germination under normal conditions to collect cotyledons and hypocotyls tissue. For these tissues, due to the small amount of material, each sample represented a pool of three young plants. Total RNA from each sample was extracted using TRIzol (Invitrogen Inc.), and the quality and integrity of extracted RNA were monitored by spectrophotometry and electrophoresis. For cDNA synthesis, 3 μg of total RNA from each sample was first treated with RNase-free DNAse I (Promega Inc.) and then reverse-transcribed using M-MLV Reverse Transcriptase (Invitrogen Inc.) and oligo-dT primers. qRT-PCR assays were performed using an ABI7500 *Real Time PCR System* (Applied Biosystems Inc.) and *SYBR® Green PCR Master Mix* (Applied Biosystems Inc.). The amplification reactions were performed using default parameters for thermal cycling (50° for 10 min, 95° for 1 min, followed by 40 cycles of 95° for 15 sec and 60° for 1 min). Primers were designed using PerlPrimer [81], attempting to choose primer pairs in which at least one of them extended across an intron-exon boundary. Expression quantification of each gene was determined according to the Ct relative quantification method (2^-ΔCt^) [82] using *SlAPT1* (adenine phosphoribosyl transferase, Solyc04g077970.2.1) as an endogenous control for data normalization. Expression data from Arabidopsis were obtained from the AtGenExpress website (jsp.weigelworld.org/expviz/expviz.jsp) [55].

## Competing interests

The authors declare that they have no competing interests.

## Authors’ contributions

The project was coordinated by EPBF. OJBB and TS performed the bioinformatics and phylogenetic analyses. AAS, MD and TS were involved in the gene expression assays. MD and TS carried out the yeast two-hybrid assays. FFS designed and performed the statistical analysis. EPBF, MD and TS prepared the manuscript. All authors read and approved the final manuscript.

## Supplementary Material

Additional file 1 **List of outgroup proteins.** Summary of the names and accession numbers of proteins used as outgroups in the phylogenetic tree of Figure 1 and Additional file 2. Click here for file

Additional file 2 **RLK Phylogenetic tree of tomato and Arabidopsis.** This is the same phylogenetic tree as presented in Figure 1, but displayed in more details. It contains additionally the accession numbers and schemes of the domain structures of each protein that composes the tree. Tomato proteins are represented by red branches and Arabidopsis proteins by blue branches. The local support values at the nodes were computed by resampling the site likelihoods 1,000 times and performing the Shimodaira-Hasegawa test. Click here for file

Additional file 3 **Expansion/reduction in Arabidopsis and tomato RLK subfamilies and functional inference.** The membership size of RLK subfamilies in Arabidopsis (At) and tomato (Sl) is indicated . Values in bold and with asterisks indicate statistical significance by the test of equal or given proportions (α=0.05). Subfamilies with significantly large proportion of duplication (dup.) or deletion (del.) were considered to have specifically expanded or reduced respectively after the divergence of Arabidopsis and tomato species. Subfamilies that presented statistically large proportion of RLKs organized in tandem repeats (t.r.) and/or of RLKs functionally annotated in defense response (def.) category were considered to be defense-related (red arrows). Conversely, subfamilies with significantly large proportion of members annotated in developmental process (dev.) category were classified as development-related (blue arrows). Green arrow indicates the LRRII subfamily that presented large proportion in both functional categories. Legend: dup.: duplication events; del.: deletion events. Click here for file

Additional file 4 **Analyses on expansion/reduction in Arabidopsis and tomato RLK subfamilies and on their functional inference.** The table contains information from Additional file 3 and presents the associated p-value from each test performed. Click here for file

Additional file 5 **List of Arabidopsis and tomato RLKs and their respective RLK subfamilies.** Summary of all RLK IDs presented in the tree of Additional file 2. Click here for file

Additional file 6 **Sequence identity between members of LRRII-RLK subfamily of tomato and Arabidopsis.** (A) Full-length amino acid sequences, (B) intracellular and (C) extracellular regions of LRRII subfamily members were aligned using CLUSTALW. Thick lines delimit the sequence comparison between members of the same clade (NIK, SERK, LRRIIc). Blue cells indicate high sequence identity, whereas red cells denote low sequence identity. Click here for file

Additional file 7 **List of primers used for yeast two-hybrid assay and for expression analysis by real-time PCR analysis.** Summary of all primers used for gene cloning and real-time PCR experiments. Click here for file
