# Supplementary figures and images for: The tomato RLK superfamily: phylogeny and functional predictions about the role of the LRRII-RLK subfamily in antiviral defense
Source: BMC Plant Biol. 2012 Dec 2;12:229. doi: 10.1186/1471-2229-12-229 (PMC3552996; doi:10.1186/1471-2229-12-229)

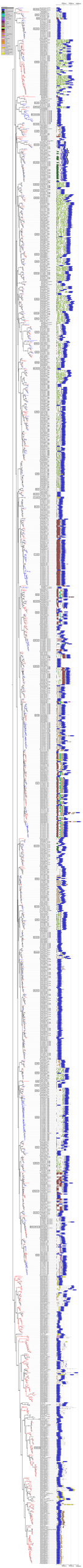

Supplement: Additional file 2 — RLK Phylogenetic tree of tomato and Arabidopsis. This is the same phylogenetic tree as presented in Figure 1, but displayed in more details. It contains additionally the accession numbers and schemes of the domain structures of each protein that composes the tree. Tomato proteins are represented by red branches and Arabidopsis proteins by blue branches. The local support values at the nodes were computed by resampling the site likelihoods 1,000 times and performing the Shimodaira-Hasegawa test. [file 1471-2229-12-229-S2.pdf]
