# Supplementary material for: The tomato RLK superfamily: phylogeny and functional predictions about the role of the LRRII-RLK subfamily in antiviral defense
Source: BMC Plant Biol. 2012 Dec 2;12:229. doi: 10.1186/1471-2229-12-229 (PMC3552996; doi:10.1186/1471-2229-12-229)
Supplement: Additional file 3 — Expansion/reduction in Arabidopsis and tomato RLK subfamilies and functional inference. The membership size of RLK subfamilies in Arabidopsis (At) and tomato (Sl) is indicated . Values in bold and with asterisks indicate statistical significance by the test of equal or given proportions (α=0.05). Subfamilies with significantly large proportion of duplication (dup.) or deletion (del.) were considered to have specifically expanded or reduced respectively after the divergence of Arabidopsis and tomato species. Subfamilies that presented statistically large proportion of RLKs organized in tandem repeats (t.r.) and/or of RLKs functionally annotated in defense response (def.) category were considered to be defense-related (red arrows). Conversely, subfamilies with significantly large proportion of members annotated in developmental process (dev.) category were classified as development-related (blue arrows). Green arrow indicates the LRRII subfamily that presented large proportion in both functional categories. Legend: dup.: duplication events; del.: deletion events. [file 1471-2229-12-229-S3.pdf]

RLK subfamilies	RLKs in At	RLKs in Sl	RLKs in ancestral	dup. in At	dup. in Sl	del. in At	del. in Sl	t.r. in At	t.r. in Sl	def. genes in At	dev. genes in At
C-LEC	1	1	1	0	0	0	0	0	0	1	1
CR4L	8	7	8	1	1	1	2	0	0	2	1
CrRLK1L-1	15	23	16	4	8	5	1	2	6	1	10*
DUF26	45	19	11	35*	10	1	2	38*	13*	20*	1
Extensin	5	6	6	0	0	1	0	0	0	0	1
L-LEC	45	23	19	30*	8	4	4	28*	4	8	4
LRK10L-2	13	14	4	12*	10*	3	0	11*	9	2	0
LysMI	2	6	4	0	2	2	0	0	2	1	0
LysMII	2	7	6	0	2	4*	1	0	2	1	0
PERK	19	19	14	9	7	4	2	5	0	1	11*
RKF3	2	2	2	0	0	0	0	0	0	0	0
SD-1	31	61	17	23*	44*	9	0	25*	45*	2	0
SD-2b	7	27	11	0	16*	4	0	0	12	1	0
SD-3	1	1	1	0	0	0	0	0	0	0	1
URK-I	2	2	2	1	0	1	0	0	0	0	0
WAK	22	15	9	18*	8	5	2	16*	9*	3	2
WAK/LRK10L-1	7	15	8	2	10	3	3	0	8*	4*	0
LRR-Ia	43	2	4	40*	0	1	2*	36*	0	8	1
LRR-Ib	2	2	2	0	0	0	0	0	0	2*	0
LRR-Ic	3	4	4	0	0	1	0	0	0	1	0
LRR-II	14	13	12	4	4	2	3	2	0	6*	8*
LRR-III	47	44	43	15	8	11	7	2	0	1	13
LRR-IV	3	2	3	1	0	1	1	0	0	0	2
LRR-IV-sis	1	1	1	0	0	0	0	0	0	0	0
LRR-V	8	9	7	2	3	1	1	0	0	0	1
LRR-VI	14	12	9	5	6	0	3	0	0	2	1
LRR-VIIa	7	8	8	1	1	2	1	0	0	0	6*
LRR-VIIb	3	2	3	0	0	0	1	0	0	1	1
LRR-VIII-1	8	8	6	3	2	1	0	3	5	0	0
LRR-VIII-2	14	19	14	6	7	6	2	10*	8	1	0
LRR-IX	4	8	7	0	3	3	2	0	0	1	1
LRR-Xa	4	6	4	1	2	1	0	0	0	2	0
LRR-Xb	10	11	12	0	0	2	1	0	0	5*	4
LRR-Xc	2	4	2	0	2	0	0	0	2	1	0
LRR-XI	26	31	29	4	7	7	5	6	6	5	21*
LRR-XII	8	48	5	5	43*	2	0	4	31*	2	0
LRR-XIIb	2	6	2	1	4	1	0	0	4	2*	0
LRR-XIIIa	4	3	3	1	0	0	0	0	0	0	3*
LRR-XIIIb	3	2	2	1	0	0	0	0	0	1	3*
LRR-XIV	3	3	3	1	0	1	0	0	0	0	2
LRR-XV	3	8	5	0	4	2	1	0	4	3*	2
RLCK-II	4	2	2	2	1	0	1	2	0	1	0
RLCK-IV	3	2	3	0	0	0	1	0	0	2	1
RLCK-V	11	8	9	4	0	2	1	0	0	1	1
RLCK-VI	14	14	12	4	3	2	1	0	0	4	0
RLCK-VIIa	45	55	40	14	20	9	5	6	12	10	4
RLCK-VIIb	1	0	1	0	0	0	1	0	0	0	1
RLCK-VIII	11	7	4	7	4	0	1	4	0	2	1
RLCK-VIII-sis	1	1	1	0	0	0	0	0	0	0	1
RLCK-IXa	2	2	3	0	0	1	1	0	0	0	0
RLCK-IXb	19	15	15	8	3	4	3	2	0	13*	2
RLCK-X	4	2	2	3	0	1	0	0	0	0	0
RLCK-XI	4	3	4	1	0	1	1	0	0	2	0
RLCK-XII	13	7	9	4	2	0	4*	2	2	1	1
RLCK-XII/XIII	12	2	1	11*	1	0	0	10*	0	1	0
RLCK-XIII	2	2	1	1	1	0	0	0	0	1	0
RLCK-XIV	1	1	1	0	0	0	0	0	0	0	0
RLCK-XV	2	3	2	1	1	1	0	0	0	0	0
RLCK-XVI	1	1	1	1	1	1	1	0	0	0	0
Total	613	631	440	287	259	114	68	129	113	214	184
